# Node Reporting and Data System 1.0 (Node-RADS) for the Assessment of Oncological Patients’ Lymph Nodes in Clinical Imaging

**DOI:** 10.3390/jcm14010263

**Published:** 2025-01-05

**Authors:** Marco Parillo, Carlo Cosimo Quattrocchi

**Affiliations:** 1 Radiology, Multizonal Unit of Rovereto and Arco, APSS Provincia Autonoma Di Trento, 38123 Trento, Italy; carlo.quattrocchi@unitn.it; 2 Centre for Medical Sciences—CISMed, University of Trento, 38122 Trento, Italy

**Keywords:** computed tomography, magnetic resonance imaging, radiology, clinical oncology, lymphatic metastasis, clinical guidelines

## Abstract

The assessment of lymph node (LN) involvement with clinical imaging is a key factor in cancer staging. Node Reporting and Data System 1.0 (Node-RADS) was introduced in 2021 as a new system specifically tailored for classifying and reporting LNs on computed tomography (CT) and magnetic resonance imaging scans. The aim of this review is to compile the scientific evidence that has emerged since the introduction of Node-RADS, with a specific focus on its diagnostic performance and reliability. Node-RADS’s performance has been evaluated in various cancer types and anatomical sites, revealing a trend where higher Node-RADS scores correspond to a greater probability of metastatic LN with better diagnostic performances compared to using short axis diameter alone. Moreover, Node-RADS exhibits encouraging diagnostic value for both Node-RADS ≥ 3 and Node-RADS ≥ 4 cutoffs in predicting metastatic LN. In terms of Node-RADS scoring reliability, preliminary studies show promising but partially conflicting results, with agreement levels, mostly between two readers, ranging from fair to almost perfect. This review highlights a wide variation in methodologies across different studies. Thus, to fully realize the potential of Node-RADS in clinical practice, future studies should comprehensively evaluate its diagnostic accuracy, category-specific malignancy rates, and inter-observer agreement. Finally, although limited, promising evidence has suggested the following: a potential prognostic role for Node-RADS; the possible value of diffusion-weighted imaging for LNs classified as Node-RADS ≥ 3; a correlation between Node-RADS and certain texture features in CT; and improved diagnostic performance when Node-RADS is integrated into radiomics or clinical models.

## 1. Introduction

The assessment of lymph node (LN) involvement is a key factor in cancer staging, as it provides valuable prognostic information and informs treatment planning. Indeed, LN status can help identify oncological patients who may be suitable for surgery vs. those who might benefit from non-surgical approaches [1]. The prevalence of LN involvement is often correlated with tumor size, stage of disease, and tumor-specific histological characteristics. Tumors with greater bulk, advanced stage, and aggressive histological features tend to have a higher risk of LN metastasis (LNM) [2].

In this context, clinical imaging modalities, such as computed tomography (CT), magnetic resonance imaging (MRI), and ultrasound, are essential for non-invasive LN assessment in routine clinical care. Superficial LNs, including those in the neck, groin, and axilla, are commonly evaluated using high-frequency linear array ultrasound transducers [3,4]. However, the limited penetration of high-frequency ultrasound restricts its application to deeper LNs, such as those within the abdomen. In these cases, lower frequency US transducers are necessary, albeit with a potential reduction in the detail visible regarding intranodal architectural changes and with limited fields of view [5]. CT and MRI offer a global view of superficial and deep LNs, allowing simultaneous evaluation with other organs during staging and follow-up examinations. While numerous studies explored the use of morphological features on CT and MRI for LN assessment, a definitive consensus on the most reliable criteria to differentiate metastatic from non-metastatic nodes is still lacking [6]. In clinical practice, LN size (for example, a short axis ≥ 10 mm) is typically used as a predictor of LNM. While LN size is often used as a screening tool, it is not a definitive indicator of metastatic disease [7]. Additionally, using LN size as a sole criterion for metastatic involvement can be inaccurate, as the normal size range of benign LNs can vary widely depending on individual factors. Moreover, a standardized approach to LN measurement, whether axial or craniocaudal, has yet to be established. Efforts to combine size and configuration criteria, as exemplified by the European Society of Gastrointestinal and Abdominal Radiology (ESGAR) consensus statement on mesorectal LNs, have shown promise in improving LN assessment [8]. Nevertheless, the specificity of these approaches to particular diseases and anatomical sites hinders their widespread clinical utility.

To enhance consistency and clarity in radiology reporting, standardized systems like Reporting and Data Systems (RADS) have gained popularity [9,10,11]. Building on this trend, Node Reporting and Data System 1.0 (Node-RADS) was introduced in 2021 as a new system specifically tailored for classifying and reporting LNs on CT and MRI scans [2]. Node-RADS aims to improve LN reporting in cancer patients by standardizing the reporting of LN characteristics; providing clear imaging criteria to increase consistency among radiologists; and being applicable to numerous cancer types and anatomical sites.

To our knowledge, this represents the first narrative review compiling the scientific evidence that has emerged since the introduction of the Node-RADS, with a specific focus on its diagnostic performance and reliability. The findings presented in this review will be instrumental in guiding future research towards those aspects of the Node-RADS score that remain less explored.

## 2. Materials and Methods

We conducted a comprehensive literature search of Scopus, Web of Science, and PubMed databases, including all articles indexed up to 29 November 2024. We used the search terms “Node-RADS” or “Node Reporting and Data System” to identify articles published in English. To ensure the highest quality of evidence, we only included original articles where Node-RADS was used with MRI or contrast-enhanced CT. Studies where Node-RADS was evaluated using other imaging modalities were excluded from our analysis (*n* = 1). We also excluded reviews (*n* = 3), editorial (*n* = 1), corrections (*n* = 3), and the original article where Node-RADS was presented (*n* = 1). After an initial review of titles and abstracts, we selected and analyzed the full text of 17 relevant articles from a pool of 26.

## 3. Node-RADS Score

**Figure 1:**
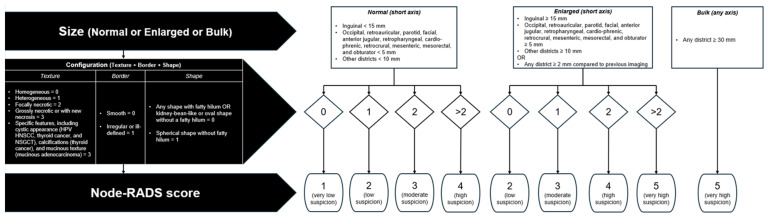
Node Reporting and Data System (Node-RADS) scoring flowchart. Modified from the original article by Elsholtz et al. [2]. First, the lymph node size is assessed and categorized as normal, enlarged, or bulky. Second, a configuration score is assigned by summing the points allocated to the features texture, border, and shape. Finally, following the flowchart, a Node-RADS score is assigned on a five-point scale (1–5), with higher scores indicating a greater likelihood of malignancy. HPV HNSCC, human papillomavirus head and neck squamous cell carcinoma. NSGCT, non-seminomatous germ cell tumors.

Node-RADS assesses LNs on a five-point scale (1–5), with higher scores indicating a greater likelihood of malignancy. The evaluation process is guided by a three-level flowchart, primarily considering LN size and configuration [2]. Node-RADS classifies LN size into three categories: normal, enlarged, and bulky. Following the initial assessment of LN size, the second-level evaluation focuses on additional anatomic features, including texture, border, and shape. The reader will give a score to each of these features and then add up the total [2]. Figure 1 summarizes the Node-RADS scoring flowchart.

Node-RADS can be applied to LN assessments on both CT and MRI. The system is flexible and does not require specific imaging acquisition parameters, making it widely applicable. While contrast-enhanced CT is essential for assessing configuration, contrast-enhanced MRI is optional due to the superior soft tissue contrast inherent to MRI [12,13]. When multiple abnormal LNs are present within a specific nodal group, the highest Node-RADS category should be used to report the overall nodal status, unless the number of metastatic nodes is a significant factor in TNM staging or treatment planning. Node-RADS categories 1 and 2 are typically reported as N(−), while categories 4 and 5 are reported as N(+). The reporting of Node-RADS category 3, which represents uncertain nodal involvement, should be determined based on the stage and histological grade of the primary tumor [2].

**Figure 2:**
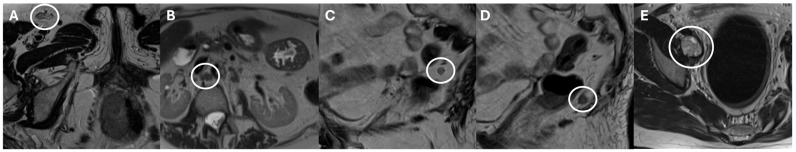
Examples of lymph nodes (LNs, circles) scored according to Node Reporting and Data System (Node-RADS) on T2-weighted images of 1.5 tesla magnetic resonance imaging. (**A**) Right inguinal LN with normal size, homogenous texture, smooth border, and preserved fatty hilum, classified as Node-RADS 1. (**B**) Interaortocaval LN with normal size, homogenous texture, irregular border, and kidney-bean-like shape, classified as Node-RADS 2. (**C**) Mesorectal LN with enlarged short axis, homogeneous texture, smooth border, and spherical shape, classified as Node-RADS 3. (**D**) Mesorectal LN with enlarged short axis, heterogenous texture, smooth border, and spherical shape, classified as Node-RADS 4. (**E**) Bulky right external iliac LN, classified as Node-RADS 5. (**A**,**C**,**D**) from a patient with anal cancer. (**B**) from a patient with breast cancer. (**E**) from a patient with rhabdomyosarcoma.

**Figure 3:**
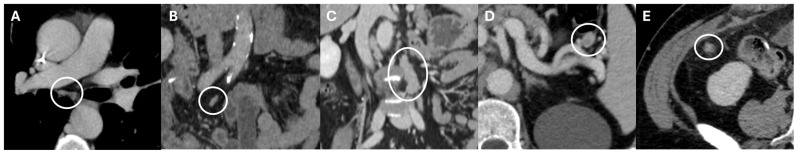
Examples of lymph nodes (LNs, circles) scored according to Node Reporting and Data System (Node-RADS) on portal venous phase of computed tomography. (**A**) Subcarinal LN with normal size, homogenous texture, smooth border, and preserved fatty hilum, classified as Node-RADS 1. (**B**) Mesenteric LN with normal size, homogenous texture, irregular border, and oval shape, classified as Node-RADS 2. (**C**) Periaortic LN with enlarged short axis, homogeneous texture, irregular border, and oval shape, classified as Node-RADS 3. (**D**) Splenic hilar LN with enlarged short axis, focal necrosis, smooth border, and oval shape, classified as Node-RADS 4. (**E**) Mesenteric LN with enlarged short axis, heterogenous texture, irregular border, and spherical shape, classified as Node-RADS 5. (**A**,**B**,**E**) from a patient with colon cancer. (**C**) from a patient with breast cancer. (**D**) from a patient with gastric cancer.

Figure 2 and Figure 3 show original examples of LNs scoring using Node-RADS on MRI and CT scans, derived from daily clinical practice.

## 4. Node-RADS’s Diagnostic Value

Since its introduction in 2021, Node-RADS’s diagnostic performance has been evaluated in various cancer types and anatomical sites. Indeed, a robust radiological classification system is expected to accurately differentiate between benign and metastatic LNs in clinical practice.

### 4.1. Neck

A retrospective study evaluated 203 cervical LNs (69% with LNM) from 119 nasopharyngeal cancer patients using Node-RADS on MRI scans. Two radiologists independently assessed the LNs. Multivariate regression analysis demonstrated that the Node-RADS score was an independent predictor of LNM. Node-RADS achieved an area under the curve (AUC) of 0.95 in diagnosing LNM. Using a Node-RADS cutoff of 3 yielded a sensitivity of 92%, specificity of 87%, positive predictive value (PPV) of 94%, and negative predictive value (NPV) of 83% [14].

Another retrospective study analyzed 1033 cervical LNs (61% with LNM) from 348 consecutive papillary thyroid carcinoma patients. Node-RADS was used to evaluate these LNs by one radiologist. The AUC for Node-RADS was 0.60. The sensitivity, specificity, PPV, and NPV were 0.68, 0.48, 0.67, and 0.50, respectively. In the same study, a group of 141 cervical LNs (72% with LNM) from 91 papillary thyroid carcinoma patients of another hospital were retrospectively evaluated using Node-RADS. The AUC for Node-RADS was 0.85. The sensitivity, specificity, PPV, and NPV were 0.85, 0.76, 0.89, and 0.68, respectively. The disparity in diagnostic performance between the two groups might be explained by the differing CT technical parameters employed at different institutions. Finally, in a prospective group of 80 papillary thyroid carcinoma patients, Node-RADS achieved an AUC of 0.73, with sensitivity of 0.67, specificity of 0.78, PPV of 0.90, and NPV of 0.44 [15].

### 4.2. Chest

A retrospective study analyzed 1134 LNs (including axillary, supraclavicular, and internal mammary LNs, of which 33% were metastatic) from 192 breast cancer patients who underwent pre-operative breast contrast-enhanced MRI and LN dissection. Three radiologists independently evaluated these LNs using Node-RADS. The AUC for Node-RADS was similar among the three readers, with values of 0.97, 0.93, and 0.93. A linear-by-linear association revealed a strong correlation between increasing Node-RADS scores and an increased risk of LNM, independent of other factors. A Node-RADS score > 2 was determined to be the optimal cutoff point, with a sensitivity of 96%, specificity of 90%, PPV of 90%, and NPV of 96% for the more experienced reader [16].

Another retrospective study of 91 lung cancer patients, including 35 with LNM, assessed the performance of Node-RADS in differentiating between benign and malignant mediastinal LNs. Two radiologists independently scored the LNs. The AUC was 0.94 for LN discrimination, without significant differences compared to the AUC using short axis diameter alone (*p* = 0.18). Moreover, a Node-RADS score threshold of 2 was found to have a sensitivity of 0.74 and a specificity of 0.93 in discriminating between benign and malignant mediastinal LNs, and even LNs categorized as Node-RADS 2 had a high malignancy rate of 43% [17].

A retrospective analysis of 173 patients with esophageal squamous cell carcinoma (49% with LNM) who underwent preoperative CT employed the Node-RADS to assess regional LNs before radical resection. The Node-RADS score demonstrated superior performance compared to individual assessment criteria, achieving an AUC of 94%, sensitivity of 96%, and specificity of 92%. Scores of 3 or higher within the Node-RADS were associated with the highest diagnostic efficacy. Notably, the diagnostic performance of the highest Node-RADS score significantly outperformed that of the short axis alone (AUC: 94% vs. 82%, *p* < 0.001) [18].

### 4.3. Abdomen

In a retrospective study, two radiologists evaluated, by consensus, regional LNs (left hilar, para-aortic, inter-aortocaval, paracaval, and right hilar) from renal cell carcinoma patients using MRI. Of the 216 enrolled patients, 58 had regional LNM. Node-RADS demonstrated superior performance to size criteria in diagnosing LNM, with a larger AUC (0.93) and higher specificity (97%) compared to size criteria (AUC of 0.88 and specificity of 87%) (*p* = 0.039 and *p* < 0.001, respectively) [19].

Another retrospective study was conducted on 91 patients with histologically proven gastric adenocarcinoma (41% with LNM). Two independent readers (a radiologist and a radiology resident) evaluated 443 LNs on CT scans using the Node-RADS classification to assess the likelihood of regional LNM. The highest diagnostic performance was achieved for Node-RADS scores ≥ 3 and ≥ 4, with sensitivity/specificity/Youden’s index of 57%/91%/0.48 and 49%/98%/0.47, respectively. The highest diagnostic performance among individual criteria was observed for a short axis diameter of 10 mm, with a sensitivity/specificity/Youden’s index of 59%/87%/0.44 [20]. Jiang et al. also retrospectively evaluated CT scans of gastric cancer patients. They analyzed 376 subjects and a total of 605 LNs (46% with LNM). The AUC for short axis diameter was 0.64. At the optimal cutoff of > 6 mm, the sensitivity and specificity were 55% and 65%, respectively, resulting in a misdiagnosis rate of 38%. A Node-RADS > 2 proved to be the most effective cutoff value, balancing sensitivity and specificity with an AUC of 0.74, sensitivity of 83%, specificity of 61%, PPV of 64%, and NPV of 81%. While increasing the cutoff to > 3 improved specificity to 86%, it significantly reduced sensitivity to 37%. Notably, 42% of LNs classified as Node-RADS 2 and 26% of LNs classified as Node-RADS 1 were histologically confirmed to be metastatic. Multivariate analysis identified Node-RADS as an independent predictor of LNM [21].

In 25 patients with perihilar cholangiocarcinoma (48% with LNM), a total of 50 hepatic hilar LNs were retrospectively assessed by two independent radiologists on CT scans. Node-RADS achieved an AUC of 0.86, similar to the AUC for short axis diameter discrimination (*p* = 0.85). A Node-RADS score threshold of 2 demonstrated a sensitivity of 78% and a specificity of 86%, with a high malignancy rate observed even in score groups 1 and 2 (42.8%) [22].

A retrospective study evaluated the clinical utility of Node-RADS in staging CT images of 108 colon cancer patients (40% with LNM). Two trained radiologists independently scored LNs, resulting in an AUC of 0.68 for LN discrimination, with no significant difference between Node-RADS score and short axis diameter (*p* = 0.85). A threshold value of 2 achieved a sensitivity of 0.62 and a specificity of 0.71 [23]. A retrospective analysis by Maggialetti et al. revealed more promising results in a similar patient population. Two radiologists independently assessed 67 patients with colon cancer (42% with LNM) on preoperative CT scans. Using the Node-RADS classification system, 95% of cases with Node-RADS scores of 1 and 2 were histologically negative, while 80% of Node-RADS 3 and 95% of Node-RADS 4 and 5 were histologically positive. Of the 28 metastatic LNs, 54% were ≥ 10 mm in size, and 46% were < 10 mm, suggesting that size alone is not a reliable predictor of negative histology [24].

Niu et al. performed two separate retrospective analyses of rectal adenocarcinoma patients, assessing mesorectal and superior rectal artery LNs on both CT and MRI scans. Two radiologists independently evaluated the imaging in both studies. In MRI scans of 154 patients (43% with LNM), Node-RADS demonstrated a good diagnostic performance with an AUC of 0.86, significantly higher than ESGAR category (AUC = 0.80, *p* = 0.04) and LN size (AUC = 0.76, *p* = 0.02). A Node-RADS score > 2 was identified as the optimal cutoff value, demonstrating a balanced performance with a sensitivity of 85%, specificity of 84%, PPV of 80%, and NPV of 87% [25]. In CT scans of 146 patients (56% with LNM), Node-RADS again significantly outperformed short axis diameter (AUC of 0.83 vs. 0.74, *p* = 0.009). Furthermore, the authors highlight that the application of Node-RADS to the prediction of LNM should focus on the LN with the largest size, unless the TNM stage or therapy depends on the number of LNs with metastasis. Notably, a Node-RADS > 3 was identified as the optimal cutoff value for CT scans, demonstrating a balanced performance with a sensitivity of 73%, specificity of 81%, PPV of 83%, and NPV of 70% [26].

A retrospective study analyzed 68 MRI scans from patients with histologically confirmed locally advanced cervical cancer (42% with LNM). Three independent radiologists assigned Node-RADS scores to pelvic LNs (common iliac, internal iliac, external iliac, and obturator). Node-RADS demonstrated strong predictive performance for nodal involvement, with an AUC of 0.94. A Node-RADS > 2 was identified as the optimal cutoff, yielding a sensitivity of 93%, specificity of 72%, PPV of 70%, and NPV of 93%. Moreover, the study demonstrated a significant positive correlation between the Node-RADS score and the presence of metastatic disease in LNs. Additionally, higher Node-RADS scores were associated with more advanced tumor stages, larger LN size, and a higher likelihood of positive surgical margins [27]. Wu et al. also retrospectively analyzed Node-RADS in MRI of cervical cancer patients. Two radiologists reviewed 729 LNs from 81 women (49% with LNM), including para-aortic, common iliac, internal iliac, external iliac, and inguinal LNs. Although there was a positive correlation between Node-RADS score and LNM rate, a surprising finding was the high rate of LNM (26.1% and 29.2%) in patients with scores 1 and 2, respectively, which contradicts the expected low probability of LNM for these scores. Additionally, a Node-RADS > 3 was found to be the optimal cutoff, demonstrating an AUC of 0.76, sensitivity of 60%, specificity of 93%, PPV of 89%, and NPV of 70% [28].

In 49 bladder cancer patients (29% with LNM), 396 regional LNs (i.e., obturator, external, internal and common iliac LNs) were retrospectively reviewed by one radiologist. Node-RADS, as assessed on CT scans, independently predicted LNM in a multivariate analysis. Additionally, Node-RADS demonstrated an AUC of 0.87 and 0.91 at the patient and LN levels, respectively. Node-RADS > 2 emerged as the optimal cutoff, balancing sensitivity of 77%, specificity of 79%, PPV of 58%, and NPV of 90% [29].

Another retrospective study evaluated 150 prostate cancer patients (24% with LNM) who underwent radical prostatectomy with extended pelvic LN dissection. Node-RADS scores were assigned to preoperative MRI scans by a single radiologist. Using a cutoff of 3 Node-Rads exhibited an AUC of 0.59 with a sensitivity, specificity, PPV, and NPV of 0.22, 0.96, 0.62, and 0.80. Considering only LNs size (<10 mm vs. ≥10 mm) at preoperative imaging as an indicator for pN+, PPV was 91% and NPV was 97%. Notably, a high proportion (20%) of LNs assigned a Node-RADS of 1–2 were found to be pathologically positive, and Node-RADS was a borderline significant predictor of LNM in multivariate analysis (*p* = 0.052) [30].

### 4.4. Summary of Evidence on Node-RADS’s Diagnostic Performance

Existing research indicates a wide variation in methodologies across different studies with sometimes conflicting results. The wide application of Node-RADS with both CT and MRI, without the standardization of technical parameters and without restrictions linked to the primary tumor, partly accounts for the heterogeneity of diagnostic performance results that emerged so far. Indeed, the reported AUC values range from a maximum of 0.97 for locoregional LNs in breast cancer to a minimum of 0.59 for pelvic LNs in prostate cancer. Our review analyzed CT and MRI studies for thirteen cancer types, and the majority of these studies were single-center and retrospective. Moreover, while Node-RADS was evaluated in ten CT and seven MRI studies, a direct head-to-head comparison of CT and MRI in the same patients and LNs was not performed in any of these studies. Thus, the effect of cancer type and imaging technique on Node-RADS diagnostic performance is not clearly understood and requires future prospective, multicenter studies with larger sample sizes. A further source of heterogeneity lies in the method used to assign Node-RADS scores to individual or multiple LNs within a nodal group. It is recommended to report the highest category among abnormal nodes, unless the number of LNM impacts staging or treatment decisions [2]. However, the included studies inconsistently reported Node-RADS scores without clear justification. In some cases, a single Node-RADS score was assigned to a patient based on the highest value obtained from the evaluation of multiple LNs (“per patient analysis”). In other cases, multiple Node-RADS scores were assigned to an individual patient based on the assessment of each individual LN (“per LN analysis”).

Overall, these studies reveal a trend where higher Node-RADS scores correspond to a greater probability of LNM. According to the Node-RADS guidelines, categories 1 and 2 should be reported as N(−), and categories 4 and 5 should be reported as N(+), while the reporting of category 3 should be tailored to the stage and histological grade of the primary tumor [2]. Node-RADS exhibited encouraging diagnostic performance for both Node-RADS ≥ 3 and Node-RADS ≥ 4 cutoffs. Although Node-RADS = 4 may identify more true positive cases, Node-RADS = 3 is more accurate in identifying true negative cases. Considering this balance, Node-RADS = 3 could be the most suitable cutoff. However, the ideal threshold for identifying LNM requires further clarification. A recent meta-analysis, including eight previously discussed studies, further confirms these results. The authors determined an AUC of 0.92 [95% confidence interval (CI): 0.89–0.94] for Node-RADS ≥ 3 and 0.91 (95% CI: 0.88–0.93) for Node-RADS ≥ 4 in diagnosing LNM. The diagnostic odds ratios (95% CI) were 31 (14–67) and 57 (23–142) for Node-RADS = 3 and Node-RADS = 4, respectively [31].

Finally, Node-RADS considering both size and configuration criteria for regional LNs could lead to better diagnostic performance compared to using short axis diameter alone, particularly in terms of specificity.

## 5. Node-RADS’s Reliability

To ensure consistent application, Node-RADS, like other RADS scores, requires validation through inter-rater reliability studies. Indeed, low inter-rater agreement could limit the system’s effectiveness in clinical practice.

The interobserver reliability between two radiologists in assessing 300 cervical LNs in MRI scans of patients with nasopharyngeal carcinoma was almost perfect (κ = 0.86 and a percentage of agreement of 79%) [14].

In a study of 1134 locoregional LNs from MRI scans of breast cancer patients, two out of three breast radiologists demonstrated substantial to almost perfect inter-observer agreement (κ = 0.71, 0.79, and 0.83) [16].

In contrast, agreement between two radiologists for Node-RADS scores in 91 mediastinal LNs from lung cancer patients was moderate (κ = 0.48) [17].

A study of 173 esophageal cancer patients undergoing CT demonstrated substantial inter-observer agreement between two radiologists in assigning Node-RADS scores (κ = 0.73) and its individual features (κ of short axis = 0.74; κ of texture = 0.75; κ of shape = 0.76; κ of border = 0.77) [18].

When assessing 216 retroperitoneal LNs in MRI scans of renal cancer patients, two abdominal radiologists and one urologist exhibited substantial interobserver agreement in Node-RADS scoring (κ = 0.75). Notably, moderate agreement was found for the assessment of texture (κ = 0.55), margin (κ = 0.42), and shape (κ = 0.51) of the LNs [19].

In 443 LNs from CT scans of gastric cancer patients, a substantial agreement was found for Node-RADS scores ≥ 3 and ≥4 (κ = 0.73 and 0.67, respectively) between a radiologist and a radiology resident. Moderate agreement was observed for texture and border contour (κ = 0.46 and 0.43, respectively), while fair agreement was found for spherical shape (κ = 0.23) [20]. In the same type of cancer patients, another study reported higher agreement between two radiologists for Node-RADS scoring of 605 LNs (almost perfect, κ = 0.86) [21].

Interobserver agreement between two radiologists in assessing 50 LNs from CT scans of cholangiocarcinoma patients was only fair (κ = 0.35) [22].

The same result was found among two trained radiologists in 108 colon cancer CT scans (κ = 0.35). Reliability was moderate for texture (κ = 0.52) and border (κ = 0.44), fair for shape (κ = 0.24), but only slight for the total configuration score (κ = 0.18) [23].

For mesorectal LNs in rectal cancer patients, substantial inter-observer agreement was found between two radiologists for both MRI (κ = 0.75) [25] and CT (κ = 0.78) [26] scans.

Inter-observer agreement between a senior radiologist and two junior radiologists for Node-RADS scoring on 68 MRI scans of cervical cancer patients was almost perfect and substantial, with κ values of 0.89 and 0.74, respectively [27]. Less encouraging results for the same type of tumor emerged from the study by Wu et al., where two radiologists classified 729 LNs, resulting an inter-observer agreement almost perfect for para-aortic LNs (κ = 0.83) but moderate for common iliac, internal iliac, and external iliac LNs (κ = 0.59, 0.55, and 0.56, respectively). The agreement for assessing individual LN features (size, texture, border, and shape) was substantial to fair (κ values of 0.68, 0.50, 0.42, and 0.36, respectively) [28].

### Summary of Evidence on Node-RADS’s Inter-Observer Reliability

A primary goal of developing Node-RADS was to standardize LN imaging reporting among radiologists. Consequently, it is crucial that Node-RADS exhibits a high degree of inter-reader agreement. It is worth noting that most current studies have limited inter-reader agreement assessments to just two observers. To fully support the widespread use of this score in clinical practice, additional research is required to evaluate agreement among multiple readers with diverse experience levels.

Available studies show mostly promising but partially conflicting results, with agreement levels ranging from fair to almost perfect for Node-RADS scoring. Notably, low agreement was observed in evaluating LN margins and shape. This may be due to the inherent subjectivity in defining irregular vs. smooth margins or in differentiating between spherical and oval nodes lacking a fatty hilum. Moreover, while the Node-RADS classification emphasizes broad applicability by omitting specific technical parameters for image acquisition, incorporating guidelines for image acquisition techniques could potentially enhance inter-observer agreement among radiologists. For instance, accurate measurements from two-dimensional images can be significantly influenced by window width, window level, and partial volume effect [32]. As window level settings impact the visualization of objects and their boundaries, it could be important to standardize this parameter by defining specific minimum, maximum, and central values. Furthermore, the accurate assessment of morphology in small LNs on MRI can be influenced by the sequence and slice thickness used. A recent study emphasized the higher reproducibility of quantitative LN measurements compared to subjective morphological assessments [33]. To enhance the reproducibility of Node-RADS, we suggest standardizing CT and MRI parameters within institutions, tailoring them to the specific tumor type. This will help minimize the impact of technical factors on scoring. Finally, it is reasonable to consider that there may be an adaptation period during the implementation of new scoring systems in clinical practice, and therefore the level of agreement among users may potentially increase with the routine integration of Node-RADS into the workflow. Additional efforts in promoting and explaining this system may be necessary for its widespread adoption.

Table 1 summarizes the main articles that have evaluated the role of the Node-RADS in clinical practice.

## 6. Other Issues

Beyond the diagnostic performance and inter-observer agreement discussed in previous sections, some studies evaluated specific aspects of Node-RADS, yielding promising but preliminary results that warrant further investigation.

### 6.1. Node-RADS’s Prognostic Role

Node-RADS is a proposed system for cancer LNs staging, which is closely linked to patient prognosis. As a relatively new classification system, long-term survival and mortality data are still being collected. In a retrospective analysis of renal cancer patients, those with a high Node-RADS score exhibited significantly worse progression-free survival (*p* < 0.001) and overall survival (*p* < 0.001) compared to patients with a low Node-RADS score during a median follow-up of 56 months. Multivariate regression analysis confirmed Node-RADS as an independent prognostic factor for renal cell carcinoma, even after adjusting for other relevant clinical variables [19].

### 6.2. Diffusion-Weighted Imaging in Node-RADS

The Node-RADS classification, primarily based on dimensional and morphological criteria, does not incorporate diffusion-weighted imaging (DWI). However, multiple studies highlighted the importance of DWI-MRI in identifying LNM without obvious central necrosis, differentiating reactive changes from neoplastic involvement, and assessing tumor spread [34,35]. In pelvic LNs of cervical cancer patients, mean apparent diffusion coefficient (ADC) values showed potential as a biomarker for predicting the status of LNs categorized as Node-RADS ≥ 3. ADC had a sensitivity of 81%, specificity of 78%, PPV of 90%, and NPV of 64%, with an AUC of 0.82 for Node-RADS ≥ 3 and 0.77 for Node-RADS 3. Optimal ADC cutoff values of 0.96 × 10^−3^ mm^2^/s were determined for both groups [27].

### 6.3. Texture Analysis and Node-RADS

Texture analysis of CT images offers a quantitative approach to assess tumor characteristics [36,37]. By analyzing the spatial patterns of CT image intensity, this technique can differentiate between malignant LNs, which often exhibit a distinct texture due to tumor cell infiltration, and benign LNs.

In hepatic hilar LNs of patients with perihilar cholangiocarcinoma, certain texture parameters correlated with Node-RADS score and specific subcategories (size, border, texture). However, shape subcategory showed no significant association with CT texture features [22].

Similar results were observed in mediastinal LNs of patients with lung cancer. Indeed, some texture parameters correlated with Node-RADS score and its subcategories, suggesting that texture analysis may offer valuable prognostic information for mediastinal LNs in lung cancer patients, potentially improving clinical decision-making [17].

### 6.4. Radiomics and Node-RADS

Radiomics is a promising field that utilizes advanced algorithms to extract quantitative information from medical images. This information can be used to develop predictive models that have the potential to improve patient outcomes and clinical decision-making [38,39].

In a cohort of gastric cancer patients, radiomics features of regional LNs on CT had an AUC of 0.75, sensitivity of 67%, specificity of 76%, PPV of 71%, and NPV of 74% in predicting LNM. A combined model that integrated radiomic features, Node-RADS classification, and traditional CT features significantly improved overall diagnostic performances when compared to models excluding radiomics, with an AUC of 0.82. In particular, subgroup analyses based on short axis diameter revealed that radiomics provided the greatest benefit for detecting LNM in small LNs (4 to 8 mm size range) [21].

### 6.5. Nomograms and Node-RADS

To fully leverage both clinical and imaging data, integrated models (i.e., clinical-imaging nomograms) could be used for preoperative evaluation of LNM.

In assessing mesorectal LNs of rectal cancer patients with MRI, a clinical-imaging nomogram that integrated tumor maximum diameter, location, carcinoembryonic antigen levels, LN short axis diameter, ESGAR category, and Node-RADS, proved to be a more powerful predictor of LNM than Node-RADS alone (AUC 0.90 vs. 0.86, *p* = 0.04) [25]. Similar results were observed using CT, where a clinical-imaging nomogram (including Node-RADS, cT stage, tumor location, and histologic grade) demonstrated significantly better performance in predicting LNM compared to Node-RADS alone (AUC 0.86 vs. 0.83, *p* < 0.001) [26].

In comparison to the validated clinical nomograms for prostate cancer MSKCC [40], Briganti 2012 [41], Gandaglia 2017 [42], and Gandaglia 2019 [43], Node RADS score 4–5 demonstrated significantly lower sensitivity (0.17 vs. 0.97, 1.00, 0.97, 0.96, respectively), significantly higher specificity (1.00 vs. 0.08, 0.10, 0.14, 0.19, respectively) and similar AUC values (0.58 vs. 0.59, 0.58, 0.57, 0.60, respectively) [30].

## 7. Conclusions

Node-RADS offers a structured approach to reporting potential LNM in cancer. There is currently a high degree of methodological heterogeneity among studies evaluating the role of Node-RADS in clinical practice, leading to sometimes conflicting findings. Overall, Node-RADS has shown promising diagnostic performance for both Node-RADS ≥ 3 and Node-RADS ≥ 4 as positive thresholds, but the optimal cutoff value for determining LNM remains uncertain. Moreover, inconsistencies in inter-observer reliability may limit its widespread clinical adoption. To fully realize Node-RADS’s potential, future studies should comprehensively assess its diagnostic accuracy, category-specific malignancy rates, and inter-observer agreement.

## Figures and Tables

**Table 1:** Main articles that have evaluated the role of the Node Reporting and Data System (Node-RADS) in clinical practice, listed in order of citation in the text. CT, computed tomography; MRI, magnetic resonance imaging; ESGAR, European Society of Gastrointestinal and Abdominal Radiology.

Investigators	Study Design	Imaging Technique	Number of Patients/Lymph Nodes	Cancer Type	Main Findings
Yang et al. [14]	Retrospective	MRI	119/203 for correlation and diagnostic performance analysis; 300 for interobserver agreement analysis	Nasopharyngeal carcinoma	Node-RADS proved to be an independent predictive factor of nodal involvement Node-RADS showed high diagnostic accuracy Node-RADS score of 3 was identified as the best cutoff for nodal involvement Almost perfect interobserver agreement in Node-RADS scoring
Yu et al. [15]	Retrospective and prospective	CT	519/1266	Papillary thyroid carcinoma	Node-RADS showed a good diagnostic performance, although lower than that of a deep learning-based automatic pipeline system
Pediconi et al. [16]	Retrospective	MRI	192/1134	Breast cancer	Node-RADS proved to be an independent predictive factor of nodal involvement Node-RADS showed high diagnostic accuracy Node-RADS score of 3 was identified as the best cutoff for nodal involvement Substantial and almost perfect interobserver agreement in Node-RADS scoring
Meyer et al. [17]	Retrospective	CT	91/91	Lung cancer	Node-RADS was effective in predicting nodal involvement for scores 4 and 5. However, high rates of nodal involvement were observed also for scores 1 and 2 A clear threshold for malignancy could not be provided by Node-RADS Texture parameters correlated with Node-RADS score Moderate interobserver agreement in Node-RADS scoring
Fang et al. [18]	Retrospective	CT	173/3550	Esophageal carcinoma	Node-RADS outperformed short diameter measurements in diagnostic performance Node-RADS score of 3 was identified as the best cutoff for nodal involvement Substantial interobserver agreement in Node-RADS scoring
Bai et al. [19]	Retrospective	MRI	216/216	Renal cell carcinoma	Node-RADS outperformed short diameter measurements in diagnostic performance High Node-RADS score has been associated with a poorer prognosis compared to low Node-RADS score Substantial interobserver agreement in Node-RADS scoring
Loch et al. [20]	Retrospective	CT	91/443	Gastric adenocarcinoma	Best performance for Node-RADS scores ≥ 3 and ≥ 4 Among individual criteria, best performance for short axis diameter of 10 mm Substantial interobserver agreement in Node-RADS scoring
Jiang et al. [21]	Retrospective	CT	376/605	Gastric cancer	Node-RADS was effective in predicting nodal involvement for scores 4 and 5. However, high rates of nodal involvement were observed also for scores 1 and 2 Node-RADS score of 3 was identified as the best cutoff for nodal involvement The integration of radiomics with Node-RADS enhanced the diagnostic performance, especially in small nodes Almost perfect interobserver agreement in Node-RADS scoring
Leonhardi et al. [22]	Retrospective	CT	28/50	Perihilar cholangiocarcinoma	No significant difference in diagnostic performance between Node-RADS and short axis measurements A clear threshold for malignancy could not be provided by Node-RADS Texture parameters correlated with Node-RADS score Fair interobserver agreement in Node-RADS scoring
Leonhardi et al. [23]	Retrospective	CT	108/108	Colon cancer	Node-RADS was effective in predicting nodal involvement for scores 4 and 5. However, high rates of nodal involvement were observed also for scores 1 and 2 A clear threshold for malignancy could not be provided by Node-RADS Fair interobserver agreement in Node-RADS scoring
Maggialetti et al. [24]	Retrospective	CT	67/67	Colon cancer	Node-RADS was correlated with nodal involvement Node-RADS score of 3 was identified as the best cutoff for nodal involvement The size criteria alone was not reliable in predicting negative histology
Niu et al. [25]	Retrospective	MRI	154/154	Rectal cancer	Node-RADS was comparable to the ESGAR category and outperformed short diameter measurements in diagnostic performance Clinical-imaging nomogram including Node-RADS had best diagnostic performance Node-RADS score of 3 was identified as the best cutoff for nodal involvement Substantial interobserver agreement in Node-RADS scoring
Niu et al. [26]	Retrospective	CT	146/292	Rectal cancer	Node-RADS outperformed short diameter measurements in diagnostic performance Clinical-imaging nomogram including Node-RADS had best diagnostic performance Node-RADS score of 4 was identified as the best cutoff for nodal involvement Substantial interobserver agreement in Node-RADS scoring
Ninkova et al. [27]	Retrospective	MRI	68/68	Cervical cancer	Node-RADS showed high diagnostic accuracy Node-RADS score of 3 was identified as the best cutoff for nodal involvement Apparent diffusion coefficient could improve diagnostic performance in Node-RADS ≥ 3 Substantial and almost perfect agreement in Node-RADS scoring
Wu et al. [28]	Retrospective	MRI	81/729	Cervical cancer	Node-RADS was effective in predicting nodal involvement for scores 4 and 5. However, high rates of nodal involvement were observed also for scores 1 and 2 Node-RADS score of 4 was identified as the best cutoff for nodal involvement Almost perfect agreement for para-aortic lymph nodes and moderate agreement for iliac lymph nodes in Node-RADS scoring
Leonardo et al. [29]	Retrospective	CT	49/396	Bladder cancer	Node-RADS proved to be an independent predictive factor of nodal involvement Node-RADS showed high diagnostic accuracy Node-RADS score of 3 was identified as the best cutoff for nodal involvement
Lucciola et al. [30]	Retrospective	MRI	150/150	Prostate cancer	Node RADS could improve specificity, but had very low sensitivity Node-RADS did not prove to be an independent predictive factor of nodal involvement A clear threshold for malignancy could not be provided by Node-RADS
